# Corn-Soy-Blend Fortified with Phosphorus to Prevent Refeeding Hypophosphatemia in Undernourished Piglets

**DOI:** 10.1371/journal.pone.0170043

**Published:** 2017-01-12

**Authors:** Anne-Louise Hother, Mikkel Lykke, Torben Martinussen, Hanne Damgaard Poulsen, Christian Mølgaard, Per Torp Sangild, André Briend, Christian Fink Hansen, Henrik Friis, Kim F. Michaelsen, Thomas Thymann

**Affiliations:** 1 Department of Nutrition, Exercise and Sports, University of Copenhagen, Frederiksberg, Denmark; 2 Department of Clinical Veterinary and Animal Sciences, University of Copenhagen, Frederiksberg, Denmark; 3 Department of Public Health, University of Copenhagen, Frederiksberg, Denmark; 4 Department of Animal Science, Aarhus University, Foulum, Denmark; 5 Department of International Health, University of Tampere, Tampere, Finland; 6 Department of Large Animal Sciences, University of Copenhagen, Frederiksberg, Denmark; US Geological Survey, UNITED STATES

## Abstract

**Background:**

Phosphorus (P) levels in refeeding diets are very important as undernourished children are at risk of hypophosphatemia during refeeding. For this reason, conventional corn-soy-blends (CSB) have been reformulated by the World Food Programme to obtain a mono-calcium-phosphate fortified product (CSB+) and a product further fortified with skim milk powder (CBS++).

**Methods:**

Using a piglet model of undernourished children, we hypothesized that feeding of CSB+, CSB++ or CSB+ with added whey permeate (CSB+/wp) would help to prevent refeeding hypophosphatemia. Pigs were weaned at 4 weeks of age and undernutrition was induced with a nutritionally inadequate pure maize diet for 7 weeks, after which they were refed for 3 weeks with either CSB+ (n = 10), CSB++ (n = 10) or CSB+/wp (n = 10). For reference, a fourth group continued on the maize diet (REF, n = 10).

**Results:**

Following induction of undernutrition, body weight and length were 29±5% and 67±4% (means±SD) of values in age-matched pigs fed a nutritionally adequate diet, and the mean serum P level was 1.77±0.34 mmol/l. During the first week of refeeding, P levels in the CSB+ pigs decreased to 55% of values before refeeding (P < 0.05) while values in the CSB++ and CSB+/wp pigs were able to maintain their plasma phosphate at a similar level as before refeeding.

**Conclusion:**

We conclude that fortification of CSB with only monocalcium-phosphate does not prevent hypophosphatemia. Dairy products like skim milk powder or whey permeate may represent relevant sources of phosphorus during refeeding. The content and form of phosphorus in such diets need to be carefully evaluated, and the undernourished piglet may be used to test the efficacy of such diets.

## Introduction

Worldwide, 50 million children suffer from acute malnutrition [1], which is a major cause of morbidity and mortality [2,3]. Food aid distribution is a key short-term approach to prevent and treat malnutrition, and corn-soy-blend (CSB) is one of the main food aid products used [4,5]. Accordingly, it has been estimated that at least 2 million moderately wasted children receive corn-soy-blend every year [5,6]. Corn-soy-blend is served as porridge and consists of precooked flour from maize and soybean grains fortified with vitamins and minerals [5]. However, recent focus on dietary management of children with moderate malnutrition and development of guidelines for recommended nutrient intakes [7] have shown that these diets may be inadequate for this target group.

Based on a recent literature review on the phosphorus status and needs, it was determined that moderately malnourished children were likely to be deficient in phosphorus and a nutrient density of 900 mg phosphorus/1000 kcal in fortified foods was proposed to allow replenishment of tissue phosphorus and compensatory growth [7]. To avoid the hypophosphatemia that may accompany nutritional rehabilitation of malnourished children, i.e. refeeding syndrome, this led to fortification of CSB with mono-calcium phosphate to compensate for the low bioavailability of organic phosphorus in plant foods. The revision of the CSB specifications by World Food Programme (WFP) led to development of two new products: CSB+ (improved corn-soy-blend for general use, fortified with phosphorus and other micronutrients) and CSB++ (improved corn-soy-blend for young and moderately malnourished children also including skimmed milk powder, sugar and oil) [5].

Skimmed milk is a good, but relatively expensive source of important nutrients like lactose, amino acids and bioavailable phosphorus [8,9]. Other dairy products like whey permeate (i.e. the remaining part of whey after removal of whey protein), could be a cheaper alternative source of lactose and bioavailable phosphorus [7,9]. Whey permeate contains very low levels of amino acids, and would therefore be inferior to skimmed milk if amino acid supply in the early refeeding phase is the most limiting factor for growth. Regardless, a prerequisite for lean body mass accretion is correction of the plasma electrolyte disturbances (i.e. low phosphate, potassium, magnesium) which are often seen in the early phase of refeeding. It is therefore important to determine how electrolyte fluctuations in the early refeeding period can be influenced via diet fortification. On this background, we hypothesized that serum phosphate levels during refeeding are better maintained when dairy products like skimmed milk powder or whey permeate are added to a corn-soy blend. Using a novel model of undernutrition in piglets [10], our study aims were: 1) to evaluate the effect of CSB+ and CSB++ on serum phosphate during refeeding, and 2) to evaluate the effect of adding whey permeate to CSB+ on serum phosphate during refeeding. The results may help to evaluate the efficacy and biological effects of refeeding-diets fortified with monocalcium phosphate and dairy products like skim milk powder and whey permeate.

## Experimental Methods

The study was approved by the *Danish Animal Experiments Inspectorate (2009/561-173 1)*, which is in accordance with the guidelines from Directive 2010/63/EU of the European Parliament.

### Animals, experimental design and diets

Forty female pigs (Duroc-x-Danish Landrace-x-Yorkshire, Høve, Denmark) were weaned at 4 weeks of age and undernutrition was induced by giving *ad libitum* access to a pure maize flour diet for 7 weeks. The degree of undernutrition following this experimental protocol, has been described in details elsewhere [10]. Following 7 weeks, the undernourished pigs were then block randomized into 4 refeeding groups based on their body weight to ensure an equal average body weight among the groups. They received 3 weeks of *ad libitum* access to either CSB+ (n = 10), CSB+/wp (n = 10), CSB++ (n = 10) or continued on the maize flour diet (REF, n = 10). Each treatment group was split into two pens with up to 5 pigs in each. The feed intake per group was recorded daily. All groups had *ad libitum* access to water throughout the study period.

The pure maize diet consisted of heat-treated maize finely ground into flour (A/S Tjørnehøj Mølle, Hedehusene, Denmark). The CSB+ and CSB++ diets were produced according to the WFP specifications for the food aid commodities at the time of production (Michiels Fabrieken, Zulte, Belgium), including precooking of the soybeans and maize grains by extrusion. Calcium carbonate (1.2% (w/w)) and sodium chloride (0.3% (w/w)) was added to CSB+ and CSB++. The CSB+/wp diet was produced on site by adding 8% (w/w) whey permeate powder (Variolac850, Arla Foods Ingredients, Denmark) to CSB+ fortified with calcium carbonate and sodium chloride (**Table 1**). The phosphorus and calcium contents were determined by inductively coupled plasma–optical emission spectroscopy (ICP-OES) (Optima 5300 DV, PerkinElmer, Waltham, MA, USA) following acidic destruction in a microwave oven (15 min at 250°C) [11]. The phytic acid content was determined on a high-performance liquid chromatography system [12].

**Table 1:** Compositions of reference and the 3 refeeding diets^1^.

	REF	CSB+	CSB+/wp	CSB++
Ingredients (g/kg (as fed))				
Maize	1000	790	730	590
Whole soybeans	-	180	160	-
De-hulled soybeans	-	-	-	180
Skimmed milk powder	-	-	-	81
Sugar	-	-	-	91
Refined soybean oil	-	-	-	30
Mineral and vitamin mix^2^	-	2.0	1.8	2.0
Monocalcium phosphate^3^	-	7.9	7.2	8.1
Potassium chloride^3^	-	7.5	6.9	7.7
Calcium carbonate^3^		12	11	12
Sodium chloride		3.0	2.7	3.0
Whey permeate^4^			81	
Composition^5^				
Energy (MJ/kg)	9.4	16	16	17
Crude protein (Nx6.25) (g/kg)	90	150	130	170
Crude Fat (g/kg)	43	62	57	91
Fiber (g/kg)	NA	25	23	20
Macrominerals (g/kg):				
Calcium	0.08	5.6	4.8	6.3
Phosphorus				
Total	4.5	4.8	4.8	5.2
Phytic acid	4.5	3.1	2.9	2.5
Non-phytic acid (% of total)	0(0)	1.7(36)	1.9 (39)	2.7 (51)
Mineral ratios				
Ca:P	0.02	1.2	1.0	1.2
Nutrient density (mg/1000 kcal)				
Phosphorus^6^	-	450	500	670

^1^CSB+ and CSB++ were produced by Michiels Fabrieken NV, (Michiels Fabrieken NV, Zulte, Belgium) according to World Food Programme (WFP) guidelines. CSB+/wp consisted of CSB + added whey permeate powder (8% (w/w)). The REF diet was produced by Tjørnehøj Mølle A/S (Tjørnehøj Mølle A/S, Hedehusene, Denmark).

^2^Vitamin/Mineral FBF-V-10 provided the following (units/kg diet when added 2 g/kg): vitamin A, 16,640 IU; thiamine, 1.28 mg; riboflavin, 4.48 mg; niacin, 48 mg; pantothenic acid, 67 mg; vitamin B6, 17 mg; folate, 600 μg, vitamin B12, 20 μg; vitamin C, 1 g; vitamin D, 40 μg; vitamin E, 83 μg; vitamin K, 1 mg; iron, 65 mg; zinc, 50 mg; and iodine, 400 μg.

^3^Monocalcium phosphate (monohydrate), potassium chloride and calcium carbonate provided nutrients (units/kg diet) when added as specified, as follows: potassium, 4 g; calcium, 5 g; and phosphorus, 2 g.

^4^Variolac®850 (Arla Foods Ingredients, Viby, Denmark) (composition: 85% lactose, 3% protein, 0.6% Na, 0.1% Mg, 0.6% P, 1.0% Cl, 1.6% K, 0.6% Ca).

^5^Energy, protein, fat and fiber analyzed by SGS (SGS, Antwerp, Belgium). Phosphorus, calcium and phytic acid contents were analyzed at the University of Copenhagen.

^6^Nutrient density for phosphorus excludes phosphorus bound in phytic acid.

### Growth and body composition

Measurements of body weight, thoracic circumference and supine length from the crown of the head to the base of the tail were recorded weekly. Body weight was measured to the nearest 0.1 kg using a digital scale, and other measurements were recorded to the nearest 0.1 cm using a standard measuring tape. These anthropometric measurements were used to assess the degree of malnutrition as described in detail below. Fat mass percentage (%Fat), bone mineral content (BMC) and bone mineral density (BMD) were determined via dual-energy X-ray absorptiometry (DXA) (QDR Explorer™, Hologic, Bedford, MA, USA) at the end of the refeeding period on anaesthetized pigs. Anaesthesia was induced with a combination of zolazepam/tiletamin (Zoletil 50 Vet, Virbac, Kolding, Denmark), xylazine (Narcoxyl 20 mg/ml, MSD Animal Health, Ballerup Denmark), ketamine (Ketaminol 100 mg/ml, MSD Animal Health, Ballerup Denmark) and butorphanol (Torbugesic 10 mg/ml, ScanVet, Fredensborg, Denmark).

### Assessment of degree of malnutrition

Classification of malnutrition in children is based on anthropometric assessment and the degree of malnutrition is classified by comparison to an international growth standard. Such reference curves are not available for pigs. Assessments of the degrees of underweight (weight relative to age), stunting (length relative to age) and wasting (weight relative to length) following 7 weeks of nutritional depletion on the maize diet were therefore based on reference growth data obtained in a trial with pigs housed under the same management and environmental conditions but fed a diet formulated to meet or exceed the nutrient requirements for pigs of this genotype [10]. The classifications of the type and severity of malnutrition were derived from the Waterlow classification used for children [13]. Based on longitudinal data on length and weight obtained from optimally nourished reference pigs [10], a prediction of weight from length was made; *weight in kg [theoretical] = e*^*3*.*77*^*kg/m x [length in meters]*^*2*.*40*^. Based on the lengths of the undernourished pigs, theoretical weights were calculated and an estimate of the degree of wasting was made by relating the theoretical weight to the observed weight (i.e. *Degree of wasting*: *(weight (observed)/weight (theoretical)) x 100%)*. The degrees of underweight and stunting following 7 weeks of nutritional depletion on the maize diet were calculated as percentages of the means of the age-matched reference pigs (i.e. *Degree of underweight*: *(weight of the undernourished pigs/mean weight of the reference pigs) x 100%; Degree of stunting*: *(length of the undernourished pigs/mean length of the reference pigs) x 100%)*. Classification of undernutrition as severe, moderate or mild-normal was done using <70%, 70–80% and >80% for weight-for-length (WFL) of the mean of the reference group, respectively [14]. Similarly, to classify the severities of stunting and underweight, <85%, 85–89% and 90–94% of the means of the references for length-for-age (LFA) and <60%, 60–75% and >75% for the means of the references for weight-for-age (WFA) were used, respectively [13,15]. Weight increment in grams per kilogram per day was estimated based on the body weight prior to initiation of refeeding.

### Blood samples

Blood samples were collected immediately before the pigs were started on the refeeding diets and then daily during the first 2 days of refeeding and after 1, 2 and 3 weeks of refeeding. The samples were taken by puncturing the *vena jugularis*, except in week 3, when the blood was collected as intracardiac samples from anaesthetized pigs just prior to euthanasia. Blood was drawn into a syringe and transferred to EDTA and serum tubes (Becton Dickinson A/S) immediately thereafter. Serum was isolated following centrifugation (2500 g, 4°C, 10 min), and the levels of albumin, phosphate, calcium, magnesium, sodium and potassium were measured using an Advia 1800 Chemistry System (quantitative, photometry, and ion selective multisensors for electrolytes, Siemens Healthcare Diagnostics, Tarrytown, NY, USA). Hemoglobin concentration in EDTA-stabilized whole blood was determined on an Advia 120 Hematology System (Siemens Healthcare Diagnostics, Tarrytown, NY, USA).

### Urine samples

Urine was collected at euthanasia by cystocentesis for assessment of urinary concentration of phosphorus and calcium. Urine was analyzed for phosphorus by the colorimetric vanadomolybdate procedure [16] and for calcium by atomic absorption spectrophotometry according to the procedure of the Association of Official Analytical Chemist (AOAC, 2000, method 975.03) [17].

### Euthanasia

Three weeks after the onset of refeeding, pigs were anesthetized with zolazepam/tiletamin (Zoletil 50 Vet, Virbac, Kolding, Denmark), xylazine (Narcoxyl 20 mg/ml, MSD Animal Health, Ballerup Denmark), ketamine (Ketaminol 100 mg/ml, MSD Animal Health, Ballerup Denmark) and butorphanol (Torbugesic 10 mg/ml, ScanVet, Fredensborg, Denmark) and then euthanized with an intra-cardiac injection of sodium pentobarbital (60 mg/kg). Samples of blood and urine were collected within 5 minutes of induction of anaesthesia. The weights of the heart, liver, kidneys, spleen, lungs, stomach, small intestine and colon were recorded.

### Statistical analysis

Data were analyzed using Stata version 12 (Stata/IC) (StataCorp LP, College Station, Texas, USA). Baseline characteristics are presented as means±SD if normally distributed or as medians (25th, 75th percentiles). The ANOVA F-test was used to evaluate differences in %Fat, BMC, BMD, organ weights, between any of the diets, and individual group differences were tested with the Sidak post-hoc test. The changes over time in weight, supine length, and serum concentrations of phosphate, magnesium, calcium and albumin were analysed using multilevel linear mixed-effects regression model (i.e. the xtmixed procedure of Stata) with pig-specific random effects, as repeated measurements per pig included in the analysis. Fixed effects included values at the start of refeeding, diet and time. Time was included as a factor variable. Model assumptions were validated and the response variables, body weight and supine length were log-transformed before analysis. Diet-time interactions were tested and included in the model if they were found to be significant (P <0.05). Post hoc comparison of the effects between the groups at different time points was performed using linear combinations of coefficients via lincom commands in STATA and adjustment for multiple comparisons when performed using Sidak. P-values lower than 0.05 were considered significant.

## Results

### Clinical characteristics

Of the 40 pigs nutritionally depleted with pure maize flour diet, 36 were refed on CSB+ (n = 9), CSB+/wp (n = 9) or CSB++ (n = 10) or continued on maize flour (REF, n = 8); 4 pigs were euthanized immediately before the onset of refeeding due to poor well-being. During refeeding, 4 out of the 36 pigs were euthanized due to severe wasting (i.e. during the first week: 1 CSB+/wp and 1 CSB++, and in the third week: 1 CSB++ and 1 REF). At the end of the 7-week nutritional depletion period, the mean body weight, supine length, and thoracic circumference of the 36 undernourished pigs were 7.6±1.4 kg, 53.4±3.5 cm, and 42.7±2.4 cm, respectively. The mean weight and length of the pigs were 29±5% and 67±4%, of the mean of the reference group, respectively. The mean WFL of the pigs was 78±10% of the mean of the reference group, respectively. Severe wasting was present in 6, moderate wasting in 14 and mild to normal wasting in 16 out of the 36 pigs. The degree of wasting before refeeding differed slightly between the groups. The % of median (interquartile range) was 77 (72, 81), 82 (79, 84), 72 (65, 75) and 84 (80, 90) for REF, CSB+, CSB+/wp and CSB++ pigs, respectively (P = 0.02). There were no differences with respect to the other measures.

The undernourished pigs were anaemic (hemoglobin <110 g/l) and hypo-albuminemic (serum albumin <35 g/l), with mean values of 58±7 g/l for hemoglobin and 19.9±4.3 g/l for albumin. The mean serum phosphate was 1.77±0.34 mmol/l. The serum magnesium was 0.69±0.10 mmol/l, and hypomagnesemia (<0.75 mmol/l) was present in 21 (66%) of the 36 pigs. The total serum calcium was 1.16±0.16 mmol/l, and serum potassium was 5.14±0.92 mmol/l.

### Feed intake

The mean daily feed intakes for the refeeding period were 346±62, 282±78 and 307±57 g/day in the CSB+, CSB+/wp and CSB++ pigs, respectively and 179±24 g/day in the REF pigs. Refeeding with either of the CSB formulations compared to REF numerically increased the feed intake of the pigs, but the difference was not tested statistically as variance could not be determined due to group housing.

### Growth and body composition

**Fig 1:**
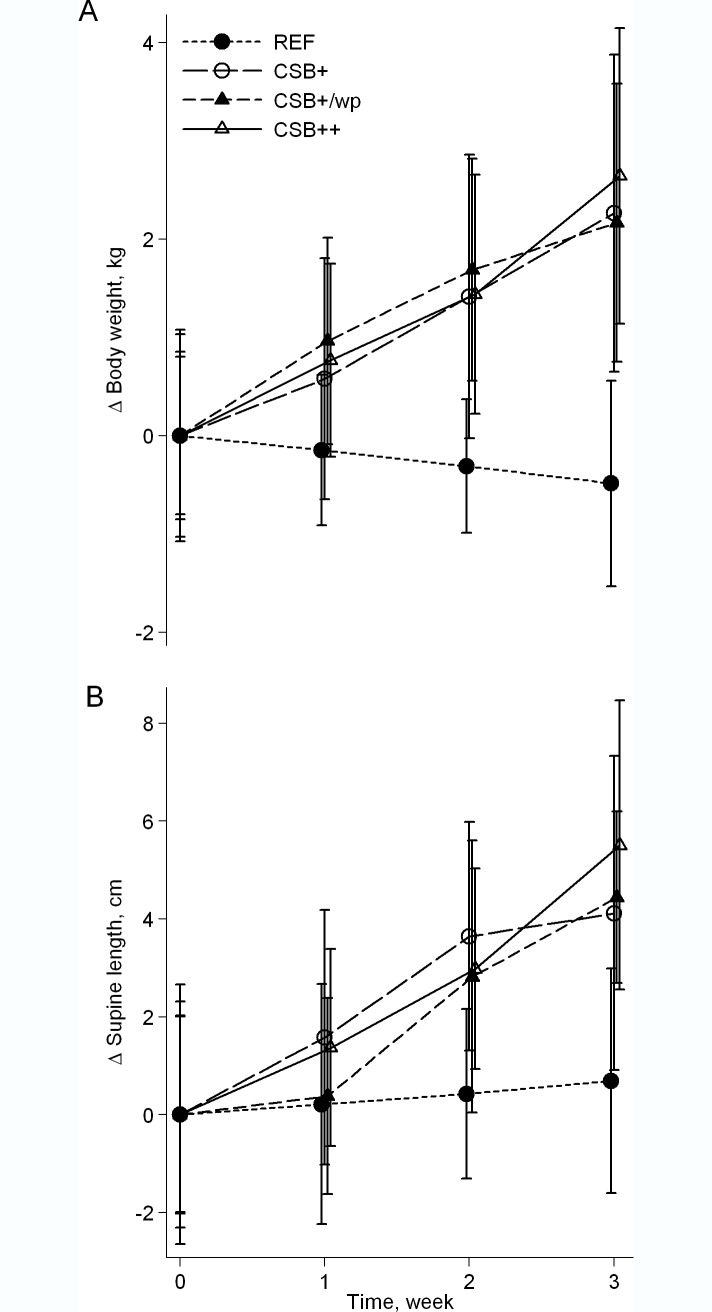
Changes in body weight (A) and supine length (B) in malnourished piglets refed with either CSB+ (n = 9), CSB+/wp (n = 9) or CSB++ (n = 10) or continuously malnourished (REF, n = 8) for 3 weeks. Values are mean (95% CI). Repeated measurement analysis was used to calculate the effects of diet (P diet), time (P time) and their interaction (P diet-time). The diet-time interaction was significant (P<0.001) for the weight increment. There was an effect of time and diet on the increment in supine length (P diet <0.01, P time <0.001). The data were log-transformed prior to analysis.

After 3 weeks of refeeding, the mean (95% CI) body weights were 34 (23, 45), 31 (21, 43) and 34 (23, 46)% higher for CSB+, CSB+/wp and CSB++ relative to REF, respectively (**Fig 1A**). Moreover, there were no differences in growth between the 3 CSB formulations. This corresponds to mean (95% CI) weight increments of 13.4 (10.5, 16.3), 12.3 (6.8, 17.7) and 13.4 (4.9, 21.8) g/kg/day for pigs fed CSB+, CSB+/wp and CSB++, respectively and -2.4 (-7.2, 2.3) for REF pigs. The mean supine lengths were 4.7 (1.9, 7.6), 3.1 (0.1, 6.1) and 5.0 (2.2, 8.0)% higher for pigs fed CSB+, CSB+/wp and CSB++ relative to REF, respectively (**Fig 1B**). There was no difference in WFL after 3 weeks of refeeding after adjustment for WFL prior to refeeding among the 3 CSB groups, whereas REF pigs were more wasted than pigs fed any of the CSB diets (REF vs pooled CSB, P = 0.007).

There were no differences in %Fat and BMD among the 4 groups after 3 weeks of refeeding (**Table 2)**. The BMC of pigs fed CSB+, CSB+/wp and CSB++ were higher than those of REF pigs. The relative weights of the liver and spleen were higher in pigs fed CSB+, CSB+/wp and CSB++ compared with REF pigs, whereas the relative lung weight was higher in REF pigs versus those fed any of the CSB formulations, although only significantly higher than CSB++. There was no effect of treatment (P>0.05) on heart, kidneys and intestine.

**Table 2:** Body composition after refeeding period of malnourished piglets with CSB+, CSB+/wp or CSB++ or continuously malnourished (REF) for 3 weeks.^1^^,^^3^.

	REF (n = 7)		CSB+ (n = 9)		CSB+/wp (n = 8)		CSB++ (n = 8)		
	Mean	95% CI	Means	95% CI	Means	95% CI	Means	95% CI	P
Body composition^2^									
% Fat (%)	13.2	12.2, 14.1	14.2	12.7, 15.6	14.4	12.7, 16.1	14.5	13.6, 15.4	0.37
BMC (g)	91.5	69.1, 114^a^	125	103, 148^b^	129	112, 146^b^	134	117, 151^b^	<0.01
BMD (g/cm^2^)	0.30	0.28, 0.32	0.33	0.30, 0.36	0.33	0.31, 0.34	0.34	0.31, 0.36	0.08
Organ weight (g/kg)									
Liver	30.9	28.4, 33.4^a^	37.1	34.4, 39.9^b^	37.5	35.6, 39.4^b^	37.4	34.1, 40.7^b^	<0.01
Spleen	3.14	2.25, 4.02^a^	5.06	4.36, 5.77^b^	5.54	4.44, 6.65^b^	4.80	3.93, 5.66^b^	<0.01
Lung	13.4	12.1, 14.6^b^	12.0	10.8, 13.3^ab^	11.9	11.1, 12.8^ab^	11.3	10.5, 12.2^a^	0.04
Heart	6.79	6.03, 7.55	6.94	6.28, 7.61	6.94	6.01, 7.87	7.53	6.89, 8.16	0.40
Kidneys	5.91	4.83, 6.99	5.81	5.44, 6.18	6.45	5.76, 7.14	6.15	5.61, 6.70	0.39
Small intestine	45.9	41.6, 50.2	48.0	43.1, 52.8	47.7	42.7, 52.8	49.8	44.3, 55.3	0.65

^1^The overall F-test for differences between any of the groups was done by ANOVA F-test, and testing of differences between each diet group to the other was done by sidak post-hoc test. Means in a row not sharing the same superscript letter, differ significantly at P<0.05.

^2^ Body composition data for CSB+ is based on n = 8, due to invalid DEXA measurements for 1 pig.

^3^Abbreviations: REF, reference diet; CSB, corn-soy-blend; CSB+, CSB *Plus*; CSB+/wp, CSB *Plus* added whey permeate; CSB++, CSB *Plus Plus*; %Fat, fat mass percentage; BMC, bone mineral content; BMD, bone mineral density.

### Serum phosphate

**Fig 2:**
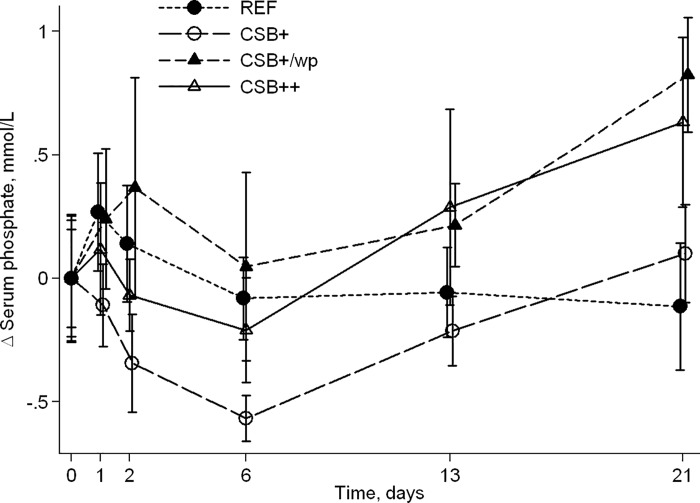
Changes in serum phosphate in malnourished piglets refed with either CSB+ (n = 9), CSB+/wp (n = 9) or CSB++ (n = 10) or continuously malnourished (REF, n = 8) for 3 weeks. Values are mean (95% CI). Repeated measurement analysis was used to calculate the effects of diet (P diet), time (P time) and their interaction (P diet-time). The diet-time interaction was significant (P <0.01).

The CSB diets had varying effects on serum phosphate over time (diet x time interaction, P <0.01) (**Fig 2**). Serum phosphate dropped during the first 48 hours in CSB+ pigs relative to REF pigs and remained low during the first week. Mean serum phosphate on day 6 after the initiation of refeeding was 1.22 (1.07, 1.38) mmol/l in the CSB+ group, corresponding to a mean (95% CI) drop in serum phosphate of 0.55 (0.39, 0.70) mmol/l. After 2 weeks of refeeding, the levels were similar to those of REF pigs. Serum phosphate in CSB+/wp and CSB++ pigs were at no time point lower than in REF pigs during the first 2 weeks of refeeding and after 3 weeks, the serum phosphate levels were 58 (30, 91)% and 47 (21, 78)% higher for pigs fed CSB+/wp and CSB++, relative to REF pigs, respectively. At the end of the refeeding period the mean serum phosphate level in pigs fed CSB++ was not different from that of the REF group, despite a tendency toward higher levels.

### Serum magnesium, calcium and albumin

Refeeding with CSB+, CSB+/wp and CSB++ resulted in increased levels of calcium and magnesium, whereas albumin remained low. There was an interaction between diet and time for calcium (P diet x time <0.01). After 1 week of refeeding, the calcium levels were higher in pigs fed any of the 3 CSB diets compared to REF and remained at the same levels during the last 2 weeks. At the end of the refeeding period, the mean serum calcium levels were 1.26±0.24 (REF), 2.06±0.13 (CSB+), 2.17±0.16 (CSB++) and 2.11±0.17 mmol/l (CSB+/wp), indicting a significantly lower level in REF relative to the three CSB groups combined (P <0.001). There was an overall positive effect of time (P time <0.01) on serum magnesium and an additional effect of CSB+, CSB+/wp and CSB++ on magnesium levels (P diet <0.001). Serum calcium increased to a level within the normal range, whereas serum magnesium was still low at the end of the refeeding period. Albumin remained low throughout the refeeding period, and after 3 weeks of refeeding serum albumin levels in CSB pigs were not different from REF pigs.

### Urine phosphorus and calcium concentration

After 3 weeks of refeeding, the pigs fed CSB+, CSB+/wp and CSB++ had lower concentrations of phosphorus in their urine compared to the REF pigs (0.814 (0.279, 1.35), 8.26 (-0.319, 16.8), 10.5 (-10.5, 31.5) and 125 (87.3, 163) mg/100g, respectively (P <0.001), whereas there were no differences among the groups for urinary calcium.

## Discussion

To our knowledge this is the first study to evaluate the effect of the improved formulations of fortified blended foods on serum phosphate. We observed a marked drop in serum phosphate during the first week of refeeding in pigs fed CSB+. A similar drop was not seen in the pigs fed maize indicating that the drop in serum phosphate was not due to malnutrition *per se*, but may instead be due to an increased demand for phosphate during refeeding, and that CSB+ was insufficient to meet this higher demand for phosphate. We found that further diet fortification with phosphorus sources like skimmed milk powder or whey permeate improves the plasma status and therefore represent diets that may potentially be useful during refeeding to improve plasma phosphate status.

Consistent with what is observed in severely malnourished children treated as inpatients [18,19], the serum phosphate nadir occurred during the first week of refeeding. In the presence of intracellular phosphate depletion, hypophosphatemia can constitute a life-threatening situation [20]. Whether the pigs had low intracellular levels of phosphate as a result of a total body deficiency of phosphorus is unknown. The very low phosphorus concentrations in the urine at the end of the refeeding period for the CSB+, CSB+/wp and CSB++ pigs compared to the REF pigs supports the conclusion that the pigs were phosphorus-depleted and that refeeding increased their need for phosphorus for growth, as individuals with very low phosphate intakes or phosphate depletion excrete minimal amount of phosphate in the urine [20,21]. However, these data should be interpreted with caution, as these concentrations are affected by diuresis, which is likely to differ between the REF and CSB pigs.

In this study, we tested the diets in a newly developed animal model of child undernutrition [10]. The nutritional status of the pigs before refeeding, as assessed by the degree of underweight, stunting and wasting mimics the scenario of a food-insecure population, in which a high level of chronic malnutrition and some degree of acute malnutrition, especially of protein and energy, are commonly observed. The pigs continuing on maize deteriorated further, and became either moderately or severely wasted. Refeeding with CSB+, CSB+/wp and CSB++ compared to REF, resulted in increased feed intake, body weight and length. All 3 CSB formulations seemed equally effective at promoting growth over a 3-week period. The weight increment corresponded to a daily gain of 12–13 g/kg^/^day, which compares well with a severely malnourished child who is treated with highly specialized diets and gains weight at a rate of 10–15 g/kg^/^day. Whether the limited effect on growth during refeeding was a result of an inadequate diet or a slow adaptation to the diet following a period of undernutrition is not clear. The limited growth observed in the initial refeeding period is in accordance with the results of other studies using refeeding diets low in protein and high in carbohydrates or a diet very similar to CSB++ [22,23]. Moreover, it is very likely that 3 weeks is too short to detect possible growth differences between the diets. The drop in serum phosphate in the CSB+ group occurred despite phosphorus fortification. Reformulation of CSB by WFP (i.e. CSB+ and CSB++) includes adding 2 g of phosphorus as mono-calcium phosphate (MCP, Ca(H_2_PO_4_)_2_) per kg of diet, corresponding to a nutrient density of approximately 500 mg/1000 kcal. Compared to the suggested nutrient density of 900 mg/1000 kcal [7] this fortification level may be too low, and the drop observed in the CSB+ group could thus be a result of an insufficient fortification. Addition of whey permeate to the CSB+ diet prevented the decline in serum phosphate either due to a slightly higher phosphorus density or a higher bioavailability of phosphorus from whey permeate compared with MCP. In a similar manner, the absence of a drop in serum phosphate in pigs fed CSB++ is either due to the higher phosphorus density or a higher bioavailability of phosphorus from milk products. The current study was designed to compare intact diets rather than the bioavailability of different phosphorus sources in malnourished individuals. Conclusions can therefore only be made on a whole diet basis and not on single nutrients.

In conclusion, the current CSB+ refeeding diet may not adequately prevent hypophosphatemia, and the content and form of dietary phosphorus should be reevaluated. Whey permeate may be a beneficial source of phosphorus when added to a base CSB diet. Regardless, safety and efficacy both short and more long term need to be shown in relevant groups of undernourished children. In perspective, if similar intracellular mechanisms between humans and this pig model can be verified, this animal model of pediatric malnutrition may help to evaluate the suitability of different refeeding diets.

## Supporting Information

S1 Data Body weight, supine length and thoracic circumference at baseline i.e. end of the 7-week nutritional depletion period. (XLS)

S2 Data Biochemical measures and mineral concentrations at baseline i.e. end of the 7-week nutritional depletion period. (XLS)

S3 Data Degree of underweight, stunting and wasting at baseline i.e.end of the 7-week nutritional depletion period. (XLS)

S4 Data Body weight measured at baseline i.e. end of the 7-week nutritional depletion period, week 1, week 2 and week 3. (XLS)

S5 Data Supine length measured at baseline i.e. end of the 7-week nutritional depletion period, week 1, week 2 and week 3. (XLS)

S6 Data Serum concentrations of phosphate, magnesium, calcium and albumin at day 0, i.e. end of the 7-week nutritional depletion period, day 1, 2, 6, 13 and 21. (XLS)

S7 Data Estimated weekly feed intake in kilograms per pig. (XLS)

S8 Data Body composition and urinary concentration of phosphorus and calcium after the refeeding period i.e. time of euthanasia. (XLS)
